# Oculomotor outcomes of cranial nerve palsy in patients with skull base tumors

**DOI:** 10.1371/journal.pone.0309686

**Published:** 2024-08-29

**Authors:** Yeji Moon, Byung Joo Lee

**Affiliations:** Department of Ophthalmology, Asan Medical Center, University of Ulsan College of Medicine, Seoul, Republic of Korea; PGIMER: Post Graduate Institute of Medical Education and Research, INDIA

## Abstract

**Objectives:**

Skull base tumors, can cause oculomotor dysfunction, presenting a management challenge given their proximity to cranial nerves. This study investigated the oculomotor outcomes in patients with skull base tumors presenting cranial nerve palsy due to tumor compression and aimed to identify associated factors.

**Methods:**

This retrospective observational cohort study enrolled patients diagnosed with primary skull base tumors who exhibited cranial nerve palsy due to tumor compression, confirmed by magnetic resonance imaging treated at Asan Medical Center between January 2011 and December 2022. Patients were assessed for oculomotor function pre- and post-treatment, and categorized into recovery and non-recovery groups based on outcomes. Factors associated with oculomotor outcomes were also analyzed.

**Results:**

Fifty-six patients were enrolled, with the majority (n = 37, 66.1%) demonstrating recovery in oculomotor function post-treatment. The duration from symptom onset to treatment initiation was short in the recovery group, suggesting that early treatment may contribute to improved oculomotor outcomes. The type of tumor was significantly associated with oculomotor outcomes, with patients with pituitary adenoma exhibiting better outcomes. In the recovery group, 19/37 (51.4%) patients underwent surgical resection alone. In contrast, in the non-recovery group, 17/19 (89.5%) patients received primary or adjuvant radiosurgery or radiation therapy.

**Conclusion:**

Approximately 70% of patients with skull base tumors experienced recovery in oculomotor function post-treatment. The duration before treatment and the type of tumor were significantly associated with the oculomotor outcome. These findings aid neuro-ophthalmologists in predicting oculomotor outcomes for patients with skull base tumors, guiding management strategies for oculomotor dysfunction.

## Introduction

Skull base tumors account for approximately one-quarter of all intracranial neoplasms [1]. These tumors can compress or infiltrate the cranial nerves along their paths, leading to oculomotor dysfunction, a common manifestation of skull base tumors. Additionally, their proximity to cranial nerves presents a significant challenge in management. Given the clinical implications of skull base tumors, neuro-ophthalmologic evaluation plays a critical role in the multidisciplinary management approach for these tumors.

Previous studies have explored oculomotor outcomes in patients with skull base tumors [2–6]. However, these studies often focused on specific types of skull base tumors, or isolated cases of cranial nerve palsy (CNP), while some included patients who developed CNP postoperatively. Postoperative CNP has a distinct etiology compared to CNP resulting from compression by the skull base tumor itself. Therefore, analyzing the clinical outcomes of CNP caused by tumor compression separately from those of cases with postoperative CNP is necessary.

The present study was conducted with the objective of determining oculomotor outcomes in patients with skull base tumors presenting with CNP due to tumor compression. Additionally, we aimed to identify factors associated with these oculomotor outcomes.

## Methods

This was a retrospective observational cohort study. The study protocol was reviewed and approved by the Institutional Review Board of Asan Medical Center (AMC IRB No. S2023-2202-0001). Moreover, all study procedures were conducted in accordance with the tenets of the Declaration of Helsinki. The requirement for informed consent was waived due to the retrospective study design.

### Study subjects

The medical records of patients who satisfied the following inclusion criteria were reviewed from 1 October 2023 to 29 February 2024, and the subject identification information has been anonymized: (1) diagnosis of a primary skull base tumor between January 2011 and December 2022, (2) treatment of the skull base tumor with medical, surgical, radiation, and/or proton therapies, (3) presence of third, fourth, or sixth CNP, as examined by a neuro-ophthalmologist before treatment, (4) confirmation of an anatomical relationship between the cranial nerve and tumor using magnetic resonance imaging (MRI), and (5) followed-up for more than 6 months after the final treatment. The exclusion criteria were as follows: (1) onset of symptoms before reaching the age of 18 years, (2) a history of strabismus surgery before the treatment for the tumor, (3) tumors originating from the sinonasal tract, nasopharynx, oropharynx, or orbit, (4) metastatic tumors, and (5) insufficient data regarding oculomotor function.

### Oculomotor function evaluations

All participants underwent a thorough evaluation of their external and internal oculomotor functions, which included assessments of duction/version, eyelid, and pupillary functions. The limitations of eye movement in each direction (adduction, abduction, supraduction, and infraduction) were quantitatively scored by a neuro-ophthalmologist, based on the severity of the limitation using a grading scale from 0 to 4. Grade 0 represented normal, full range of eye movement, while Grade 4 signified complete palsy. Grades 1 to 3 were defined by increments of 25% limitation in eye movement for each grade. Eyelid function was evaluated on a three-level scale: Grade 0 for normal function, Grade 1 for partial ptosis, and Grade 2 for complete ptosis. Pupillary function assessment was categorized as Grade 0 for normal response and Grade 1 when a diminished light reflex or presence of anisocoria was observed. Finally, "severe oculomotor impairment" was defined as Grade 3 to 4 limitation in eye movement, or complete ptosis during the pre-treatment examination.

The outcomes of oculomotor function were divided into two groups based on comparisons between pre-treatment and final neuro-ophthalmologic examinations: the recovery (RC) group and the non-recovery (NC) group. The RC group included patients who demonstrated an improvement of one grade or more in eye movement, eyelid, or pupillary function, without worsening in any aspect of oculomotor function. The NC group comprised patients who either demonstrated no change or experienced a progressive deterioration in eye movement, eyelid, or pupillary function, with an escalation of one grade or more in any oculomotor function.

### Statistical analysis

All data with continuous variables are presented as means ± standard deviations, while categorical data are presented as proportions and percentages. Mann–Whitney U test and Fisher’s exact test were applied to identify the factors correlated with oculomotor outcomes. Statistical significance was set at *p*-values < 0.05. All statistical analyses were performed using SPSS version 23.0 (IBM Corp., Armonk, NY, USA).

## Results

### Basic demographic and clinical characteristics

A total of 56 patients were included in the final analysis (Table 1 and S1 File). The mean age at the symptom onset was 54.2 ± 12.5 years, and females represented 53.6% of the study population. The mean follow-up period was 1.6 ± 1.5 years, and the average duration from symptom onset to treatment initiation was 10.4 ± 20.7 (median 3.6, range 0.1–120.6) months. The most common presentation was isolated sixth CNP, observed in 32 patients (57.1%), followed by isolated third CNP in 17 patients (30.4%). Furthermore, six patients (10.7%) presented with multiple CNPs. Severe oculomotor impairment was noted in 26 patients (46.4%).

**Table 1:** Baseline demographic and clinical characteristics of the study population.

Characteristics	Total (n = 56)	Recovery (n = 37)	Non-recovery (n = 19)	*p*-value
Age, years	54.2 ± 12.5	53.5 ± 13.5	55.5 ± 10.7	0.709
Female sex, n (%)	30 (53.6)	20 (54.1)	10 (52.6)	> 0.999
Follow-up period, years	1.6 ± 1.5	1.5 ± 1.4	1.9 ± 1.7	0.094
The period from symptom onset to treatment, months	10.4 ± 20.7	4.2 ± 5.4	22.1 ± 31.7	**0.001**
Cranial nerve involvement, n (%)				0.810
Isolated oculomotor nerve (CNIII)	17 (30.4)	11 (29.7)	6 (31.6)	
Isolated trochlear nerve (CNIV)	1 (1.8)	1 (2.7)	0 (0.0)	
Isolated abducens nerve (CNVI)	32 (57.1)	22 (59.5)	10 (52.6)	
Multiple nerves	6 (10.7)	3 (8.1)	3 (15.8)	
Severe oculomotor impairment, n (%)	26 (46.4)	16 (43.2)	10 (52.6)	0.578
Intracranial tumor, n(%)				**0.029**
Meningioma	23 (41.1)	10 (27.0)	13 (68.4)	
Pituitary adenoma*	17 (30.4)	15 (40.5)	2 (10.5)	
Chordoma	7 (12.5)	5 (13.5)	2 (10.5)	
Chondrosarcoma	3 (5.4)	1 (2.7)	2 (10.5)	
Schwannoma	2 (3.6)	2 (5.4)		
Hemangioma	2 (3.6)	2 (5.4)		
Germ cell tumor	1 (1.8)	1 (2.7)		
Epidermoid cyst	1 (1.8)	1 (2.7)		
Treatment for tumor, n (%)				**0.003**
Surgery	20 (35.7)	19 (51.4)	1 (5.3)	
Surgery + adjuvant therapy	11 (19.6)	6 (16.2)	5 (26.3)	
Gamma knife radiosurgery	4	3	1	
Fractionated radiotherapy	6	3	3	
Proton therapy	1		1	
Gamma knife radiosurgery	12 (21.4)	6 (16.2)	6 (31.6)	
Cyberknife radiosurgery	12 (21.4)	6 (16.2)	6 (31.6)	
Fractionated radiotherapy	1 (1.8)	0 (0.0)	1 (5.3)	

CN, cranial nerve.

* Including eight patients with pituitary apoplexy.

Meningioma was the most prevalent tumor type, identified in 23 cases (41.1%), including 13 patients with cavernous sinus meningiomas, and 10 with petroclival meningioma. In 20 patients, meningioma originated from or invaded the cavernous sinus. Pathologic diagnosis post-surgery confirmed the type of meningioma in eight patients: three with World Health Organization (WHO) Grade I, four with WHO Grade II, and one with WHO Grade III meningioma.

Pituitary adenoma was diagnosed in 17 patients (30.4%), eight of whom experienced pituitary apoplexy. Chordoma was identified in seven patients (12.5%), while three patients (5.4%) were diagnosed with chondrosarcoma. Patients with schwannoma (n = 2), hemangioma (n = 2), germ cell tumor (n = 1), and epidermoid cyst (n = 1) were also enrolled in the study.

Surgical resection of the tumor was performed in 31 patients (55.4%), with 11 receiving adjuvant therapy. The remaining 25 patients received treatments other than surgical resection: 12 underwent Gamma Knife radiosurgery, 12 received Cyberknife radiosurgery, and one was treated with fractionated radiotherapy.

### Oculomotor outcomes

Among the total cohort, 37 (66.1%) patients achieved recovery of their oculomotor function (RC group), with 33 of these patients achieving complete recovery to Grade 0 across all measures of oculomotor function. Conversely, 19 patients (33.9%) were categorized into the NC group, with seven patients in this group demonstrating a progressive worsening of oculomotor function by one grade or more.

Table 1 presents the clinical characteristics according to oculomotor outcomes, revealing no significant difference in age or sex between the two groups. However, the NC group experienced a notable prolonged duration from symptom onset to the initiation of their first treatment (*p* = 0.001). Further, we identified no significant correlation between the pattern of cranial nerve palsy (third, fourth, sixth, or multiple CNPs) or the presence of severe oculomotor impairment and the final oculomotor outcome.

Importantly, the type of tumor was significantly associated with oculomotor outcomes (*p* = 0.029). Among patients with meningioma, 13 out of 23 (56.5%) exhibited persistent or worsening CNP, whereas 15 out of 17 (88.2%) patients with pituitary adenoma demonstrated recovery of their oculomotor function following treatment. All patients diagnosed with schwannoma, hemangioma, germ cell tumor, or epidermoid cysts demonstrated complete recovery post-treatment.

In terms of treatment modality, more than half of all patients in the RC group (19 of 37 patients, 51.4%) underwent only surgical resection for tumor treatment. In contrast, within the NC group, surgery alone was the treatment of choice for only one patient (5.3%), and 17 of 19 (89.5%) patients received primary or adjuvant radiosurgery or fractionated radiotherapy. The difference in treatment modality demonstrated statistical significance (*p* = 0.003).

Additional subgroup analyses were performed on patients with meningioma and pituitary adenoma. (Table 2) In the meningioma group, the duration from symptom onset to treatment was 9.9 ± 7.1 months in the RC group and 29.8 ± 36.0 months in the NC group. In the pituitary adenoma group, the duration was 1.3 ± 1.3 months in the RC group and 3.7 ± 3.2 months in the NC group. However, these differences did not reach statistical significance (*p* = 0.277 and *p* = 0.184 respectively).

**Table 2:** Analysis of factors associated with oculomotor outcomes according to the types of tumors.

Characteristics	Meningioma (n = 23)			Pituitary adenoma (n = 17)		
	Recovery (n = 10)	Non-recovery (n = 13)	*p*-value	Recovery (n = 15)	Non-recovery (n = 2)	*p*-value
Age, years	57.1 ± 9.9	55.6 ± 6.4	0.574	56.0 ± 13.8	53.6 ± 20.0	0.956
Female sex, n (%)	9 (90.0)	8 (61.5)	0.179	3 (20.0)	1 (50.0)	0.426
Follow-up period, years	1.3 ± 0.8	2.2 ± 2.0	0.211	1.9 ± 1.7	1.8 ± 1.1	0.662
The period from symptom onset to treatment, months	9.9 ± 7.1	29.8 ± 36.0	0.277	1.3 ± 1.3	3.7 ± 3.2	0.184
Cranial nerve involvement, n (%)			0.855			0.603
Isolated oculomotor nerve (CNIII)	3 (30.0)	4 (30.8)		7 (46.7)	2 (100.0)	
Isolated trochlear nerve (CNIV)	0 (0.0)	0 (0.0)		0 (0.0)	0 (0.0)	
Isolated abducens nerve (CNVI)	6 (60.0)	6 (46.2)		6 (40.0)	0 (0.0)	
Multiple nerves	1 (10.0)	3 (23.1)		2 (13.3)	0 (0.0)	
Severe oculomotor impairment, n (%)	4 (40.0)	7 (53.8)	0.680	9 (60.0)	1 (50.0)	> 0.999
Treatment for tumor, n (%)			0.813			**0.044**
Surgery	0 (0.0)	1 (7.7)		13 (86.7)	0 (0.0)	
Surgery + adjuvant therapy	4 (40.0)	3 (23.1)		0 (0.0)	1 (50.0)	
Gamma knife radiosurgery	2	1		0	0	
Fractionated radiotherapy	2	2		0	1	
Proton therapy	0	0		0	0	
Gamma knife radiosurgery	2 (20.0)	5 (38.5)		1 (6.7)	1 (50.0)	
Cyberknife radiosurgery	4 (40.0)	3 (23.1)		1 (6.7)	0 (0.0)	
Fractionated radiotherapy	0 (0.0)	1 (7.7)		0 (0.0)	0 (0.0)	

CN, cranial nerve.

There was no significant effect of the type or severity of palsy on oculomotor outcomes in either disease group. In the meningioma group, there was no difference in treatment modality between the RC and NC groups. However, in the pituitary adenoma group, 86.7% of patients in the RC group underwent only surgical resection, while the two patients in the NC group received primary or adjuvant gamma knife radiosurgery. This difference in treatment modality was significant in the pituitary adenoma group (*p* = 0.044).

## Discussion

In this study, 66% of patients experienced complete or partial recovery from their CNP, with 60% achieving full recovery of their oculomotor function. Notably, the RC group had a shorter duration from symptom onset to the first treatment than that in the NC group. This correlation between the duration before treatment and cranial nerve recovery aligns with findings from several prior studies, suggesting that prolonged symptoms before treatment may lead to irreversible cranial nerve damage due to sustained compression by the tumor [7–9]. In the subgroup analysis, meningioma showed a longer duration of symptoms before treatment than pituitary adenoma. Meningioma tends to cause prolonged CNP compared to pituitary adenoma, which may partially contribute to this correlation [6]. However, the duration of symptoms before treatment was shorter in the RC group than in the NC group for each disease type, although statistical significance could not be demonstrated due to the small sample size. Therefore, prompt radiological investigation and management are critical for patients presenting with new-onset cranial neuropathy.

Our analysis demonstrated that the type of tumor significantly influenced oculomotor outcomes, with pituitary adenomas associated with better recovery compared to meningiomas, corroborating findings from previous research [2,6]. In our cohort, eight patients with pituitary apoplexy who underwent surgical resection within a month of symptom onset achieved complete recovery of their oculomotor function, potentially impacting the study’s results. When excluding patients with pituitary apoplexy, the duration from symptom onset to the first treatment remained significantly associated with oculomotor outcomes (*p* = 0.017). However, the association between the type of tumor and the oculomotor outcomes lost statistical significance (*p* = 0.230). Apoplexy could, therefore, contribute to favorable oculomotor outcomes in patients with pituitary adenoma. A sudden increase in pressure due to pituitary apoplexy can cause CNP by compressing the nerve or damaging to vasa nervorum, so the decompression can restore oculomotor function, resulting in favorable oculomotor outcomes [10,11].

Interestingly, patients with schwannoma, hemangioma, germ cell tumor, and epidermoid cyst, who constituted a minority of the total study participants, all demonstrated good outcomes, with complete recovery of oculomotor function. Two patients with cavernous sinus hemangioma underwent radiosurgery, and the remaining four patients underwent surgical resection. In contrast, 13/23 patients (56.5%) with meningioma were classified into the NC group, indicating less favorable oculomotor outcomes for meningioma compared to those of other skull base tumors. Previous studies have demonstrated variable recovery rates for cranial nerve function following meningioma treatment, depending on the tumor’s location, WHO grade, and treatment modality [5,6,12–16]. In this study, most patients with meningioma (n = 20, 87.0%) demonstrated the involvement of cavernous sinus. Furthermore, among the eight cases where WHO grade classification was possible, five were identified as WHO grade 2 or 3 meningiomas. Accordingly, most of the patients with meningioma in this study were treated with primary (n = 15) or adjuvant (n = 7) radiosurgery or fractionated radiotherapy, which may have led to a significant difference in the type of treatment between the RC and NC groups when analyzing entire patient population.

However, there was no effect of treatment modality on the oculomotor outcomes in the meningioma group. Previously Li-Pei et al. also reported that treatment did not influence recovery rates in the meningioma group [6]. To determine the effect of treatment modality on oculomotor outcome in meningioma patients, further systematic investigations considering tumor size, WHO grade, and exact location, in particular, cavernous sinus invasion should be performed.

Chordoma and chondrosarcoma are rare, locally aggressive tumors that occupy the same anatomical location and have very similar clinical and radiological presentations [17]. In several previous studies involving patients who underwent surgical resection for chordoma and chondrosarcoma, chordoma was suggested to exhibit more aggressive biological behavior, causing severe disability compared to chondrosarcoma [18,19]. In this study, two of seven patients with chordoma and two of three patients with chondrosarcoma were classified into the NC group. However, the comparison of oculomotor outcomes between the two disease groups could not be analyzed owing to the small number of patients. Furthermore, pathologic diagnosis could not be confirmed in 3/7 patients with chordoma and 2/3 patients with chondrosarcoma, as they underwent radiosurgery without surgical resection.

This study’s limitations include its small sample size, particularly the low number of patients with each type of skull base tumor, excluding pituitary adenoma and meningioma. This limitation, unfortunately, restricted our ability to comprehensively analyze oculomotor outcomes for each tumor type and precluded a multivariable analysis, potentially weakening the statistical significance of our findings. Further research involving a large cohort and multivariable models is required to accurately identify factors associated with oculomotor outcomes. Second, as we included the patients with skull base tumors who received various treatments depending on the judgment of the clinician and the patient’s condition, our ability to compare the results of specific treatments is limited. Last, tumor size, exact location, and growth patterns on MRI were not included in this analysis.

## Conclusions

Despite the aforementioned limitations, this study uniquely evaluates oculomotor outcomes from a neuro-ophthalmologist’s perspective across various skull base tumors, concluding that approximately 70% of patients achieve recovery of oculomotor function post-treatment. The duration before treatment and the type of tumor were both significantly associated with the oculomotor outcome. Our findings can offer valuable insights for neuro-ophthalmologists in predicting patient prognosis and planning future treatment strategies.

## Supporting information

S1 File Data from patients included in this study. (XLSX)
